# Multi-omics study reveals gut microbiota dysbiosis and tryptophan metabolism alterations in GH-PitNET progression

**DOI:** 10.1038/s41598-025-07812-x

**Published:** 2025-07-07

**Authors:** Jifang Liu, Zhang Ye, Yi Zhang, Shen You, Siqi Sun, Lin Lu, Wan Su, Jie Liu, Jun Pu, Hui Pan, Huijuan Zhu, Kan Deng, Yong Yao, Xiaomin Hu, Shuyang Zhang

**Affiliations:** 1 Department of Neurosurgery, Peking Union Medical College Hospital, Chinese Academy of Medical Sciences and Peking Union Medical College, Beijing, 100730 China; 2 Pituitary Disease Innovative Diagnosis and Treatment Center, Peking Union Medical College Hospital, Chinese Academy of Medical Sciences and Peking Union Medical College, Beijing, 100730 China; 3 Biomedical Engineering Facility of National Infrastructure for Translational Medicine, Peking Union Medical College Hospital, Chinese Academy of Medical Sciences and Peking Union Medical College, Beijing, 100730 China; 4 Department of Endocrinology, Peking Union Medical College Hospital, Chinese Academy of Medical Sciences and Peking Union Medical College, Beijing, 100730 China; 5 State Key Laboratory of Complex Severe and Rare Diseases, Peking Union Medical College Hospital, Chinese Academy of Medical Science and Peking Union Medical College, Beijing, 100730 China

**Keywords:** GH-PitNETs, Gut microbiota, Indole-3-acetic acid, Bacterial pathogenesis, Biomarkers

## Abstract

Growth hormone-secreting pituitary neuroendocrine tumors (GH-PitNETs) arise from the anterior pituitary gland and constitute 20–30% of all PitNETs, representing a significant subset of functional pituitary tumors. Despite their prevalence, the precise mechanisms underlying the development of these tumors remain elusive due to the complex pathophysiology of pituitary neoplasia. To investigate the potential role of the gut microbiome in GH-PitNETs, we conducted a comprehensive study involving 16S rRNA gene sequencing and metabolomics analysis of fecal and serum samples from 20 GH-PitNET patients and 30 healthy controls at Peking Union Medical College Hospital. Our findings revealed a distinct gut microbiota profile in GH-PitNET patients compared to healthy individuals, characterized by dysbiosis with increased abundance of *Bacteroides* and decreased abundance of *Blautia* and *Bifidobacterium*. Notably, alterations in specific bacterial taxa, including *Intestinibacter bartlettii*, *Fusicatenibacter faecihominis*, and *Massilioclostridium*, were observed in GH-PitNET patients. Concomitantly, serum metabolomics analysis identified 154 differentially abundant metabolites in GH-PitNET patients, with significant enrichment in pathways related to tryptophan metabolism. Among these metabolites, 3-indoleacetic acid (IAA) exhibited a obvious change, suggesting its potential research value for disease processing of GH-PitNETs. To further elucidate the mechanistic link between the gut microbiome and GH-PitNETs, we conducted in vitro and in vivo experiments, our results demonstrated that IAA could promote the proliferation of GH3 cells and significantly enhance growth hormone secretion by activating the cAMP pathway. These findings collectively suggest that gut microbiota dysbiosis may contribute to in the development and progression of GH-PitNETs by contributing to metabolic disturbances.

**Supplementary Information:**

The online version contains supplementary material available at 10.1038/s41598-025-07812-x.

## Introduction

Pituitary neuroendocrine tumors (PitNETs) constitute 16% (11–22%) of intracranial tumors and represent a common and significant disease in neurosurgery^1^. Growth hormone-secreting PitNETs (GH-PitNETs), a functional pituitary tumor, originates from the anterior pituitary lobe and accounts for 20–30% of PitNETs^2^. In addition to causing tumor compression symptoms such as headache, vomiting, blurred vision, and visual field defects, GH-PitNETs leads to endocrine and metabolic disorders due to the long-term excessive secretion of growth hormone and insulin-like growth factor-1 (IGF-1), resulting in conditions such as acromegaly in adults and gigantism in children^3,4^.

The pathogenesis of GH-PitNETs is multifaceted, involving both genetic and environmental factors^5–13^. A growing body of research suggests that genetic mutations, particularly in the *METTL3* gene, play a significant role in the development of these tumors^14^. These mutations activate the *GNAS* and *GADD45γ* genes, thereby promoting tumor growth and growth hormone secretion. Other factors, including genetic predispositions, growth factors, and cytokines, may also contribute to the initiation and progression of GH-PitNETs^15–17^. However, the precise mechanisms underlying the occurrence and development of GH-PitNETs remain incompletely understood.

Numerous studies have reported on the impact of microbial metabolites on various diseases, including the identification of variations in GH-PitNETs bacteria that may contribute to disease pathogenesis. However, the specific mechanisms underlying these associations and the role of metabolites as potential mediators remain unclear^5,18^. The diversity of the host’s bacterial community is influenced by factors such as diet and physiological status. This recognition highlights the microbiome and metabolome as promising non-invasive targets for precision medicine^18–20^. While a few studies have explored the associations between the gut microbiota and GH-PitNETs, a systematic investigation of the interplay between the gut microbiota, serum metabolites, and their impact on GH-PitNETs is lacking^18^. Emerging evidence suggests that the intestinal microbiota can influence solid tumor development through the secretion of specific metabolites that modulate tumor cell metabolism and growth or by altering the tumor microenvironment^21^. Consequently, disruptions in the intestinal flora balance and dysregulation of specific serum metabolites may contribute to tumorigenesis^22–30^. However, the complex interplay between the gut microbiota, serum metabolites, and their role in the development of GH-PitNETs remains understudied.

**Fig. 1:**
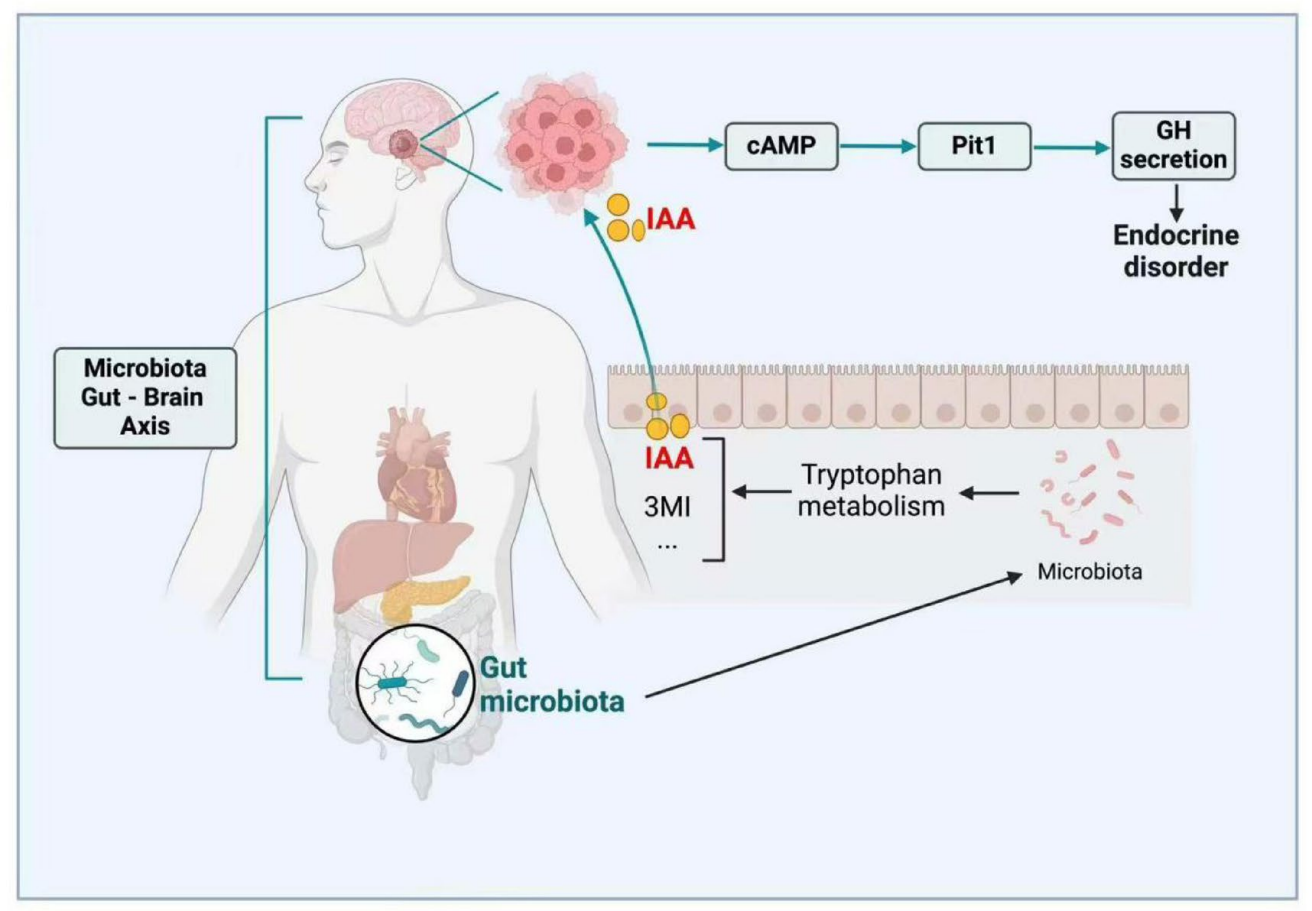
Gut dysbiosis elevates IAA, activating cAMP to drive GH secretion and tumor progression in GH-PitNETs.

Therefore, we designed this study to recruit 20 GH-PitNET patients and 30 age- and sex-matched healthy control (HC) for a comprehensive quantitative analysis of the microbiome and serum metabolome of fecal samples from human GH-PitNET patients and healthy control, with the aim of looking for differential microbes and differential metabolites between GH-PitNET patients and HC. Finally, the relationship between GH-PitNETs and intestinal microbiota and serum metabolites was interpreted, and our study proved the relationship between bacteria and tryptophan metabolism and growth hormone secretion. The tryptophan-derived metabolite IAA may modulate the cAMP signaling pathway, potentially facilitating GH secretion, as outlined in Fig. 1.

## Materials and methods

### Study design and sample collection

The study, including all experimental procedures, was approved by the Ethics Committee of Peking Union Medical College Hospital, adhering to the guidelines of the Council for International Organizations of Medical Sciences (Ethics approval number: I-24PJ0158). All participants provided written informed consent prior to inclusion in the study, ensuring they were fully aware of the study’s aims, procedures, potential risks, and benefits. Ethical guidelines were strictly followed, including confidentiality of participant information and the option to withdraw from the study at any time without penalty.

In this study, a sample of 20 GH-PitNET patients and 30 controls was selected based on preliminary power calculations to ensure sufficient statistical power. Using G*Power software, we performed a two-tailed independent t-test with an assumed effect size of 0.9, reflecting a large effect based on similar studies. We set the significance level at 0.05 for a two-tailed test and aimed for a power level of 0.8. The analysis indicated that a minimum of 20 patients per group would be necessary to detect meaningful differences. To further account for variability and enhance the robustness of our findings, we included 20 GH-PitNET patients and 30 healthy controls. The formulas used are as follows:

$$n=\frac{2\times {({Z}_{\frac{\alpha }{2}}+{Z}_{\beta })}^{2}}{{d}^{2}}$$

where Zα/2 is the Z-value corresponding to the significance level (α). For a two-tailed test at α = 0.05, Zα/2 = 1.96; Zβ is the Z-value corresponding to the power level (1−β). For a power of 0.8, Zβ = 0.84; d = 0.9, the anticipated effect size (Cohen’s d).

Between January 2023 and June 2023, a cohort of 20 patients diagnosed with sporadic GH-PitNETs and 30 healthy control (HC) volunteers were recruited from Peking Union Medical College Hospital (Beijing, China). These individuals underwent routine medical investigations, including pituitary plain magnetic resonance imaging (MRI) and dynamic contrast-enhanced MRI. Prior to their inclusion in the study, written informed consent was obtained from all GH-PitNET patients and healthy volunteers. The researchers meticulously recorded the patients’ general clinical data, encompassing disease course (CD), age, sex, body mass index (BMI), Knosp classification, history of obstructive sleep apnea syndrome (OSAS), history of diabetes mellitus (DM), history of hypertension (HTN), growth hormone nadir during the oral glucose tolerance test (OGTT-GH nadir), GH level, and p53 and Ki67 immunohistochemical staining results. Notably, all GH-PitNET patients and volunteers were of Han Chinese ethnicity and shared similar geographic regions and dietary habits. The definitive diagnosis of GH-PitNETs in all patients was established through postoperative pathologic examination. To ensure the integrity of the study, several exclusion criteria were applied: patients aged 80 years or older or younger than 25 years, patients with multiple endocrine neoplasia type 1 (MEN1), patients who had undergone preoperative radiotherapy or octreotide treatment, patients with concurrent tumors, and patients with metastatic PitNETs. Healthy volunteers were selected based on the criteria of normal bowel habits and the absence of a history of tumors or other serious gastrointestinal diseases. Additionally, both patients and volunteers who had used antibiotics, probiotics, prebiotics, or symbiotics within two months prior to sample collection were excluded. To facilitate further experiments, all eligible fecal samples were self-collected by participants and promptly transported to the laboratory. Upon arrival, each sample was divided into three aliquots, packed into separate freezer tubes, and stored at − 80 °C.

### Reagents

The antibodies employed in this study included anti-phospho-ERK1/2 (Thr202/Tyr204) (Proteintech, 28733-1-AP), anti-ERK1/2 (Proteintech, 11257-1-AP), anti-phospho-CREB1 (Ser133) (Proteintech, 28792-1-AP), anti-CREB1 (Proteintech, 12208-1-AP), anti-Pit1 (Abcam, ab313642), anti-AHR (Proteintech, 67785-1-lg) and anti-β-actin (Proteintech, 66009-1-Ig). The reagents utilized in this study were 3-indoleacetic acid (IAA) (10 mM, dissolved in DMSO, MCE, HY-18569) and forskolin (1 mM, dissolved in DMSO, MCE, HY-15371).

### Cell culture

The GH3 cell line, a rat pituitary tumor clone that synthesizes and secretes prolactin and growth hormone, was used for in vitro studies. The cell line was purchased from the National Infrastructure of Cell Line Resources (Beijing, China) and was certified by the institute. GH3 cells were cultured in DMEM supplemented with 10% fetal bovine serum (FBS) and 1% penicillin (50 U/ml)/streptomycin (50 mg/ml) in an incubator at 37 °C in a humidified environment with 5% CO_2_.

### In vivo experiment

Male C57 mice, 5 weeks old, were used for this experiment, housed in a controlled environment at 22 ± 2 °C with a 12-h light/dark cycle, with ad libitum access to food and water. Mice were randomly divided into two groups: a treatment group receiving indole-3-acetic acid (IAA) at a dose of 100 mg/kg (p.o.) and a control group receiving an equal volume of saline, both administered by oral gavage once daily for seven consecutive days. On the eighth day, blood was collected from the retro-orbital sinus under anesthesia and centrifuged at 3000 rpm for 10 min to separate the serum, which was then stored at − 80 °C until ELISA analysis. This experiment was approved by the Animal Welfare Ethics Committee of Beijing MDKN Biotechnology Co., LTD., and was conducted in strict accordance with the experimental animal care and use guidelines of Beijing Animal Control Committee.

### Extraction of fecal DNA

All patient samples were collected during the active disease phase, specifically at the time of diagnosis, prior to any treatment interventions. First, fecal samples are homogenized in a lysis buffer containing SDS, proteinase K, and bead-beating to mechanically disrupt the cells. After incubation, the sample undergoes phenol–chloroform extraction, followed by a purification step using a silica column to isolate DNA. To assess DNA quality, we measure the A260/A280 ratio to check purity and run an agarose gel to verify integrity. We also include a final wash step to remove contaminants that might affect subsequent PCR or sequencing processes. Subsequently, the DNA was diluted to a concentration of 1 ng/µL using double-distilled water.

### 16S ribosomal RNA gene sequencing

A specific, barcoded primer pair was used to amplify the 16S rRNA gene from distinct regions. PCR reactions were performed using 15 µL of Phusion® High-Fidelity PCR Master Mix (New England Biolabs), 2 µM of each forward and reverse primer, and 10 ng of template DNA. PCR products were mixed with an equal volume of loading dye containing SYBR Green and visualized on a 2% agarose gel. Subsequently, the PCR products were pooled and purified using a Qiagen Gel Extraction Kit (Qiagen, Germany). A TruSeq® DNA PCR-Free Sample Preparation Kit (Illumina, USA) was employed to generate sequencing libraries following the manufacturer’s protocol. Library quality was assessed using a Qubit® 2.0 Fluorometer (Thermo Fisher Scientific) and an Agilent Bioanalyzer 2100 System. Finally, the library was sequenced on an Illumina NovaSeq platform to generate 250 bp paired-end reads.

### Analysis of sequencing data

Paired-end reads were demultiplexed according to their unique barcodes, assigning each read to its originating sample. Subsequently, the paired-end reads were merged using FLASH (version 1.2.7). Quality filtering was performed using QIIME (version 1.9.1) to generate high-quality, clean sequence tags. These tags were compared against the Silva reference database (https://www.arb-silva.de/). Vsearch (https://github.com/torognes/vsearch/) was used to identify and remove chimeric sequences, resulting in a set of effective clean tags. Sequence analysis was performed using UPARSE (version 7.0.1001). Sequences with a similarity greater than 97% were clustered into operational taxonomic units (ASVs). A representative sequence from each ASV was used for annotation with the Silva Database. Alpha diversity, including the Shannon and Simpson indices, was calculated for the fecal samples using QIIME (version 1.9.0). Beta diversity analysis was also performed using QIIME (version 1.9.0). The Mann–Whitney U test was used to assess differences in alpha diversity between groups, with a significance level set at *p* < 0.05. The Bray–Curtis distance matrix was calculated using the ade4 R package for Principal Coordinates Analysis (PCoA), and the ggplot2 R package was used to generate the sorted plots. Permutational Multivariate Analysis of Variance (PERMANOVA) was employed to compare beta diversity. For comparing the differential Operational Taxonomic Units (OTUs) between groups, the Student’s t-test was utilized to evaluate the differences in OTUs, with a significance level set at *p* < 0.05. The Benjamini–Hochberg method was applied for *p*-value correction to identify significant OTUs. OTUs with significantly increased or decreased abundance were displayed in a volcano plot based on fold-change and *p*-value. Differences in the abundance of significant OTUs across different groups were shown in boxplots, all visualized using the ggplot2 R package. Using SPSS software, we calculated the Spearman correlation between OTUs and clinical indicators along with the corresponding *p*-values, and a heatmap was created for visualization using the heatmapR package.

### Metabolite extraction from blood samples

One hundred microliters of blood were added to Eppendorf tubes and mixed with pre-chilled 80% methanol using a vortex mixer. The mixture was then incubated on ice for 5 min, followed by centrifugation at 15,000 × g at 4 °C for 20 min. A portion of the supernatant was diluted with liquid chromatography–mass spectrometry (LC‒MS)-grade water to achieve a final methanol concentration of 53%. The diluted samples were transferred to new Eppendorf tubes and centrifuged under the same conditions.

### Acquisition of LC–MS data

Analytical procedures were conducted using a Vanquish UHPLC system coupled with an Orbitrap Q Exactive HF mass spectrometer, both from Thermo Fisher Scientific (Germany), at Novogene Co., Ltd. in Beijing. Samples were introduced onto a Hypesil Gold column (100 × 2.1 mm, 1.9 μm) and separated using a 17-min linear gradient at a flow rate of 0.2 mL/min. For positive polarity, a mobile phase consisting of 0.1% formic acid in water (A) and methanol (B) was employed, while for negative polarity, 5 mM ammonium acetate (pH 9.0) in water (A) and methanol (B) were used. The gradient program involved an initial 2% B hold for 1.5 min, a linear increase to 85% B over 3 min, a ramp to 100% B over 10 min, a decrease to 2% B in 10.1 min, and a final 12-min hold at 2% B. The mass spectrometer was operated in both positive and negative ion modes with the following settings: spray voltage of 3.5 kV, capillary temperature of 320 °C, sheath gas flow rate of 35 psi, auxiliary gas flow rate of 10 L/min, S-lens RF level of 60, and auxiliary gas heater temperature of 350 °C.

### Analysis of LC‒MS-based metabolomics

UHPLC-MS/MS raw data underwent processing using Compound Discoverer 3.1 (Thermo Fisher) for peak alignment, selection, and metabolite quantitation. Parameters included a retention time tolerance of 0.2 min, a mass tolerance of 5 ppm, a 30% tolerance for signal intensity, a minimum signal-to-noise ratio of 3, and a specified minimum peak intensity. Following peak intensity normalization to total spectral intensity, molecular formula prediction was facilitated by considering additive ions, molecular ion peaks, and fragment ions. mzCloud (https://www.mzcloud.org/), mzVault, and the MassList database were then employed for metabolite identification and relative quantification. Statistical analysis, excluding compounds with a relative peak area coefficient of variation (CV) exceeding 30% in quality control (QC) samples, was performed using R (version 3.4.3), Python (version 2.7.6), and CentOS (version 6.6). Finally, metabolite identification and relative quantification were achieved, with annotations drawn from KEGG (https://www.genome.jp/kegg/pathway.html), HMDB (https://hmdb.ca/metabolites), and LIPIDMaps (http://www.lipidmaps.org/) databases. ROC curves for relevant serum metabolites were calculated using metaboanalyst (https://www.metaboanalyst.ca/) and visualized with pROC [1.18.0] and ggplot2 [3.3.6]. Additionally, Spearman correlation analysis was employed to assess correlations between serum metabolites and clinical indicators, with results visualized as heatmaps generated by ggplot2 [3.3.6].

### CellTiter-Glo (CTG) proliferation assay

CTG proliferation assays were conducted in accordance with the manufacturer’s protocol. Briefly, 5000 cells per well were seeded in 96-well plates. On the following day, cells were treated with varying concentrations of IAA. After a three-day incubation period, CTG reagent (Promega) was added to lyse the cells. Luminescence was subsequently measured using a BioTek Synergy H1 Plate Reader (BioTek, Winooski, VT).

### EdU cell proliferation assay

GH3 cells were seeded in six-well plates and treated with varying concentrations of IAA for 24 h to assess cell proliferation via 5-ethynyl-2′-deoxyuridine (EdU) incorporation assay. Subsequently, cells were incubated with EdU for 2 h. Following fixation with 4% paraformaldehyde and permeabilization with 0.3% Triton X-100, cells were incubated with a pre-prepared Click-iT reaction buffer for 30 min. Finally, nuclei were counterstained with DAPI and visualized using a Cytation 7 imaging system (BioTek Instruments, Winooski, VT, USA).

### Growth hormone measurement

Growth hormone (GH) secretion was quantified using a Rat GH ELISA Kit and a Mice GH ELISA Kit (Elabscience Biotechnology Co., Ltd.) according to the manufacturer’s protocol. Cells were resuspended in PBS containing 1% protease inhibitor, subjected to ultrasonic disruption, and centrifuged at 1000 × g for 20 min. The supernatant was collected for GH measurement. Absorbance was detected at 450 nm using a BioTek Synergy H1 Plate Reader. Protein concentration was determined using a BCA protein quantification kit (Beyotime Biotechnology) for normalization. GH in GH3 cell supernatant was assayed using a Rat GH ELISA Kit (Elabscience). Supernatants were centrifuged (1000 × g, 20 min), aliquoted, and stored at − 80 °C. Samples and standards were loaded in triplicate onto pre-coated plates. After incubation, detection antibody, HRP-streptavidin, and TMB substrate were added sequentially. Absorbance (450 nm) was measured (BioTek Synergy H1).

### Western blotting assay

The collected cells were lysed in RIPA buffer supplemented with 1% phosphatase and protease inhibitors and incubated on ice for 30 min. Following centrifugation at 4 °C and 12,000 × g for 15 min, protein concentration in the supernatant was determined using a BCA protein quantification kit (Beyotime Biotechnology, Shanghai, China). Equal amounts of protein was separated by SDS-PAGE, transferred to PVDF membranes, and probed with a specific antibody. Target protein bands were visualized using a Bio-Rad chemiluminescence imager (Hercules, CA, USA) and quantified by densitometry analysis with ImageJ.

### RNA-sequence

Total RNA was extracted from corresponding treated GH3 cells using TRIzol reagent (Invitrogen). RNA quality and quantity were assessed with an Agilent Bioanalyzer and a Qubit RNA Assay Kit (Invitrogen), respectively. Poly(A) mRNA was enriched using magnetic beads to remove ribosomal RNA. RNA-seq libraries were prepared with the TruSeq RNA Library Prep Kit (Illumina), quantified via qPCR, pooled, and sequenced on an Illumina HiSeq 2500 platform to generate 150 bp paired-end reads. Data quality was assessed using FastQC, and sequences were aligned to the reference genome using the STAR aligner. Gene expression was quantified using HTSeq-count, and differential expression analysis was performed using DESeq2. RNA Integrity Numbers (RIN) for each sample are as follows:

**Table:** 

	CON1	CON2	CON3	IAA1	IAA2	IAA3
RIN	9.4	9.5	9.7	9.6	9.6	9.4

### Statistical analysis and visualization

In the analysis of experimental data, all data were presented as mean ± standard deviation (SD). Student’s t-test was used to determine whether there were statistically significant differences between groups, with a significance level set at *p* < 0.05. Statistical results were visualized using GraphPad software. For the analysis of omics data, several R packages were utilized, including ggraph, ggClusterNet, Hmisc, and igraph, to calculate the correlations between bacteria and metabolites and to construct their respective co-abundance networks. Additionally, the igraph package was used to compute the correlations between key bacteria, metabolites, and clinical indicators. The node and edge files were imported into Cytoscape software to visualize the correlation network among the three entities. The remaining visualizations were generated using RStudio.

## Results

### Characteristics of the research cohort

In this investigation, a cohort of individuals with GH (n = 20) and a control group of HC (n = 30) were enrolled. To mitigate dietary variability, participants were selected based on similar dietary patterns. A comprehensive assessment of clinicopathological variables (Table 1) confirmed comparable characteristics between the two groups, eliminating potential confounding factors. As depicted in Table 1, GH-PitNET patients exhibited elevated levels of Ki67 and P53, indicative of pituitary tumor activity. A significant disparity in body mass index (BMI) was observed, with GH-PitNET patients (27.16 ± 2.959 kg/m^2^) exhibiting a higher BMI than HC participants (23.36 ± 2.633 kg/m^2^) (*p* < 0.0001). Furthermore, the prevalence of diabetes mellitus was significantly higher in the GH-PitNETs group (35%) compared to the HC group (0%) (*p* = 0.0008), likely attributable to the underlying endocrine disorder.

**Table 1:** Clinicopathological factors of the GH-PitNET patients and healthy volunteers.

Characteristics	Value for group				*p* value
	Healthy subjects		GH-PitNET patients		
All cases	30		20		
Age	43.7 ± 10.0		47.1 ± 14.3		0.4456
Gender					0.3662
Female	22 (73.3%)		12 (60%)		
Male	8 (26.7%)		8 (40%)		
General obesity (BMI) (kg/m^2^)	23.36 ± 2.633		27.16 ± 2.959		< 0.0001
Hypertension					0.2484
Negative	12 (40%)		12 (60%)		
Positive	18 (60%)		8 (40%)		
Diabetes mellitus					0.0008
Negative	30 (100%)		13 (65%)		
Positive	0 (0)		7 (35%)		
Ki67					NA
< 3%	NA		15 (75%)		
≥ 3%	NA		5 (25%)		
P53					NA
Negative	NA		13 (65%)		
Positive	NA		7 (35%)		
Knosp					NA
0	NA		10 (50%)		
1	NA		2 (10%)		
2	NA		3 (15%)		
3	NA		3 (15%)		
4	NA		2 (10%)		
nadir GH during OGTT (ng/ml)	NA		5.366 (1.700–24.600)		NA

BMI, body mass index; NA, not available.

Data are presented as mean (range) unless otherwise specified.

### Overview of the gut microbiome in the HC and GH groups

**Fig. 2:**
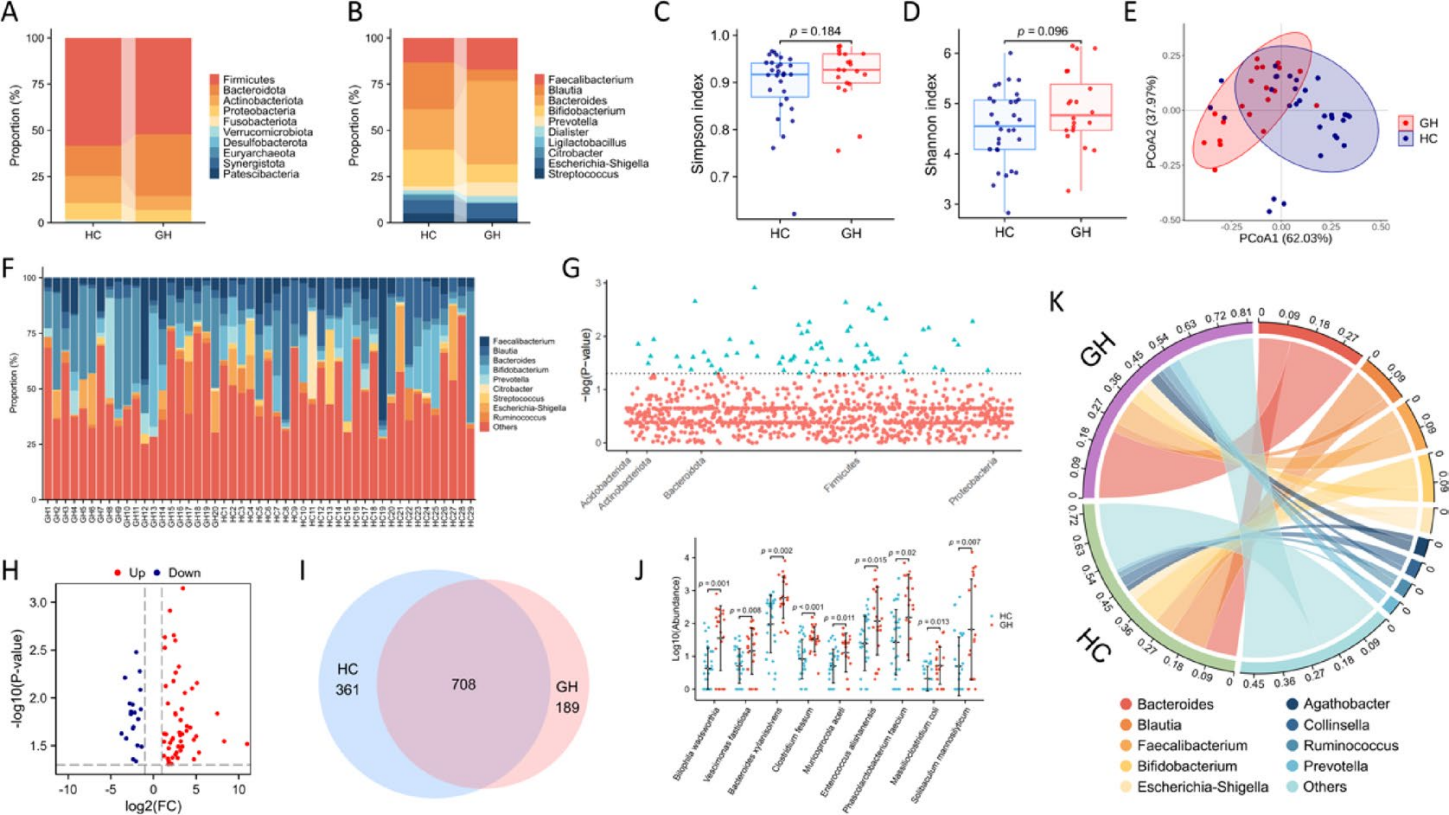
Overview of the gut microbiome in the HC group (n = 30) and the GH group (n = 20). (**A**) Dominant phyla in each group. (**B**) Dominant genera in each group. Alpha diversity differences between the HC and GH groups were estimated by the Simpson (**C**) and Shannon (**D**) indices. (**E**) Differences in beta diversity between the HC and GH groups. (**F**) Component proportions of bacterial genera in each subject. (**G**) The distribution of differential ASVs at the phylum level. (**H**) Differential ASVs between the HC and GH groups. (**I**) The number of ASVs in each group. (**J**) The nine ASVs with the most significant differences in abundance between the HC and GH groups. (**K**) Dominant genera and their contributions to each group.

In this study, we analyzed a comprehensive dataset of 3,895,199 high-quality 16S rRNA reads, yielding a median of 77,084 reads per sample, ranging from 63,321 to 92,841. After rigorous denoising, 1461 distinct ASVs were identified. Analysis of the relative abundance of dominant phyla revealed ten primary phyla in each group (Fig. 2A). *Firmicutes* represented the most prevalent phylum, comprising 58.3% of the HC group and 52.3% of the GH group. *Bacteroidota* was the second most abundant phylum, constituting 14.6% of the HC group and 7.5% of the GH group. Other notable phyla included *Proteobacteria*, *Fusobacteriota*, and *Actinobacteria*. At the genus level, ten primary genera were identified in each group (Fig. 2B). Bacteroides was the most prevalent genus, accounting for 23.2% of the GH group and 10.8% of the HC group. Other dominant genera included *Faecalibacterium*, *Blautia*, *Bifidobacterium*, and *Prevotella*. To assess bacterial diversity, alpha and beta diversity metrics were calculated based on sequence alignments. The Simpson and Shannon indices (Fig. 2C and D, respectively) did not reveal significant differences between the HC and GH groups (*p* = 0.096 and *p* = 0.184). However, weighted PCoA indicated a distinct separation between the two groups based on the first two principal components (Fig. 2E), suggesting a potential influence of tumor burden on gut microbiota diversity, although further investigation is needed to confirm this association. Microbial taxon assignment was employed to assess the relative abundance of dominant genera in both groups. This analysis revealed notable inter-individual variability in gut microbiota composition within each group (Fig. 2F). Manhattan plots were generated to visualize the contribution of differentially abundant ASVs at the phylum level (Fig. 2G). The relative abundance of these ASVs is depicted in a volcano plot (Fig. 2H). A Venn diagram was utilized to analyze the presence of ASVs with a relative abundance exceeding 0.1% across different groups (Fig. 2I). The nine ASVs exhibiting the most significant differences in abundance between the HC and GH groups are highlighted in Fig. 2J. Figure 2K illustrates the predominant genera at the genus level and their respective contributions to each group.

### Relationship between the gut microbiota and clinical phenotype in patients with GH

**Fig. 3:**
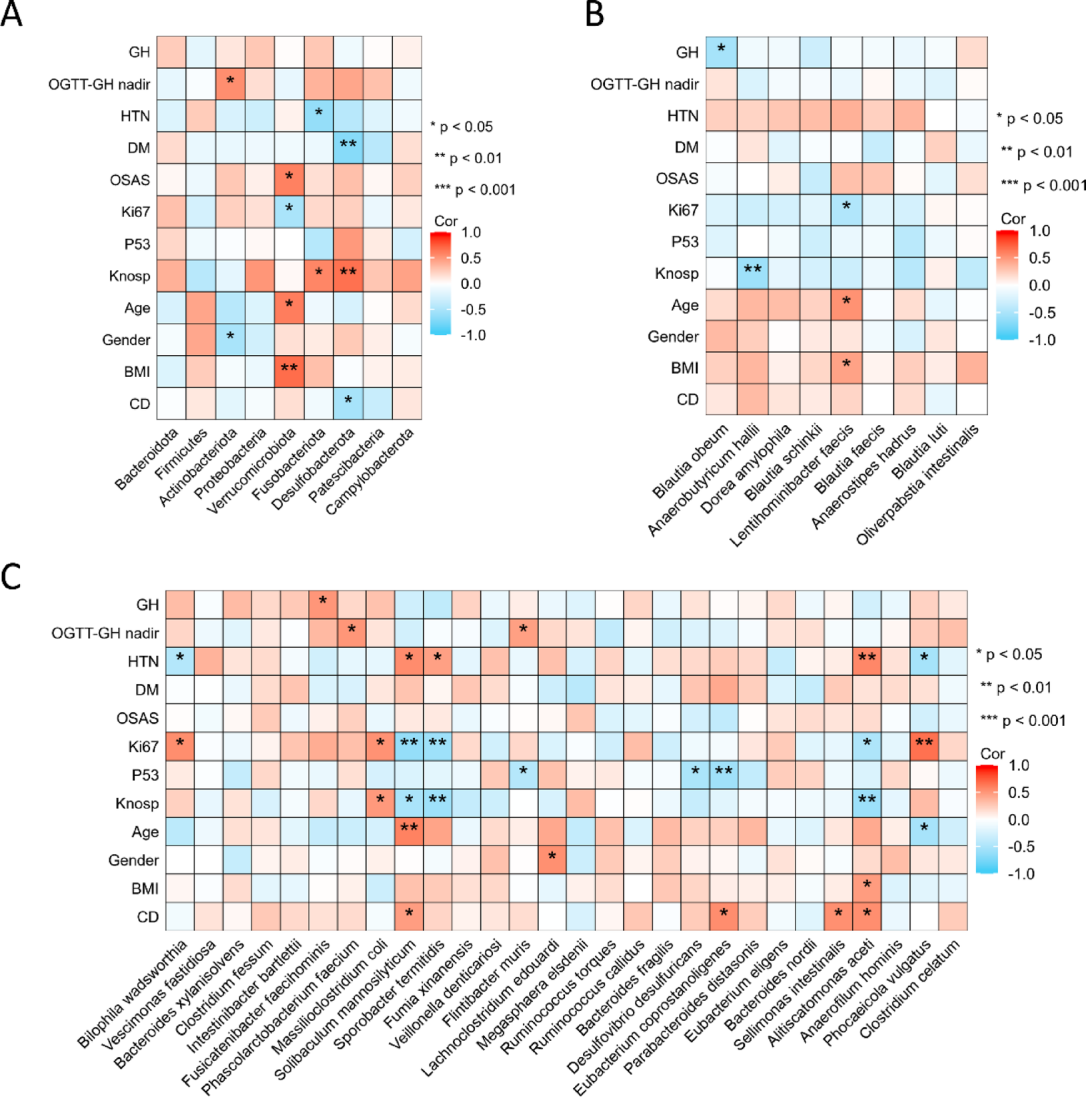
Flora-clinical correlation analysis revealed that the presence of GH-positive and HC-positive bacteria was associated with the severity of the clinical symptoms of GH-PitNETs. (**A**) Bacterial phyla associated with GH-PitNETs clinical symptom severity. (**B**) HC-positive bacterial phyla correlated with GH-PitNETs clinical symptom severity. (**C**) GH-positive bacterial genera associated with GH-PitNETs clinical symptom severity.

Next, we investigated the associations between gut microbiota diversity and clinical phenotypes, focusing on disease severity and complications. Correlations were analyzed at both the phylum and genus levels. At the phylum level, five phyla were significantly correlated with various indicators of disease severity, including GH levels, nadir GH levels during the OGTT, Ki67 and p53 staining, and Knosp grade (Fig. 3A). Notably, four of these nine differential phyla, namely *Actinobacteriota*, *Verrucomicrobiota*, *Fusobacteriota*, and *Desmoplasia*, exhibited positive correlations with these severity markers. Among the genera enriched in healthy controls, five were significantly associated with increased disease severity and complications. Interestingly, four out of nine of these genera displayed negative correlations (Fig. 3B). Conversely, within the genera enriched in GH-PitNET patients, 16 were significantly associated with disease severity and complications. Approximately 35% (11 out of 31) of these genera showed positive correlations, including *Intestinibacter bartlettii*, *Fusicatenibacter faecihominis*, and *Massilioclostridium coli*, with *Intestinibacter bartlettii* being particularly prominent. This analysis revealed distinct patterns of correlation between specific genera and disease outcomes (Fig. 3C). These findings suggest a potential link between specific gut microbiota and the progression or exacerbation of GH-PitNETs.

### Changes in serum metabolites in GH-PitNET patients

**Fig. 4:**
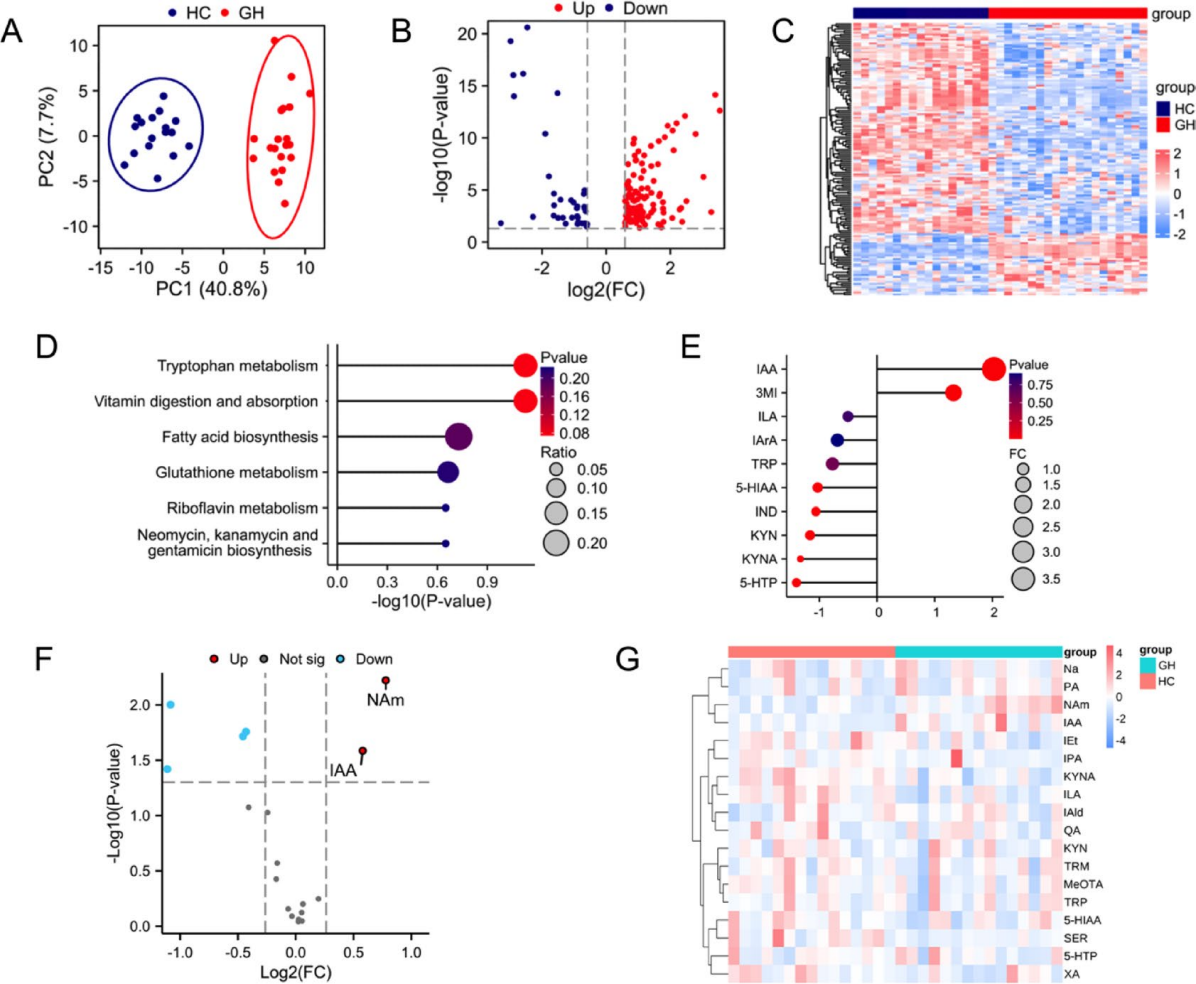
Abnormal tryptophan metabolism in the intestinal flora of GH-PitNET patients. (**A**) OPLS-DA modeling revealed significant differences in the serum metabolic profiles between the GH and HC groups. (**B**) Volcano plot showing the differential distribution of serum metabolites between the GH and HC groups. (**C**) Heatmap showing the differential distribution of serum metabolites between the GH and HC groups. (**D**) Metabolite enrichment in tryptophan metabolism pathways via MetaboAnalyst (https://www.metaboAnalyst.ca/). (**E**) Variable importance in projection (VIP) values of serum tryptophan metabolites and their ratios of biological significance. (**F**) Volcano plot showing differentially abundant metabolites that target tryptophan metabolism. (**G**) Heatmaps showing the changes in metabolites that target tryptophan metabolism in the GH group and HC group.

PCA was employed to analyze differences between the GH and HC groups (n = 20 and n = 17, respectively), revealing group-specific metabolomic profiles (Fig. 4A). We investigated serum metabolites that exhibited significant differences between these groups, as illustrated in Fig. 4B,C. The analysis indicated that the differentially accumulated metabolites primarily participate in tryptophan metabolism. Other affected pathways included vitamin digestion and absorption, fatty acid biosynthesis, glutathione metabolism, and riboflavin metabolism (Fig. 4D). Within the tryptophan metabolic pathway, we identified differentially abundant metabolites. As shown in Fig. 4E, IAA and 3-methylindole (3MI) were significantly elevated in the blood of GH-PitNET patients. Consequently, we hypothesized that IAA and 3MI may be positively correlated with GH levels. To further validate this hypothesis, we conducted targeted tryptophan metabolomics analysis on a subset of samples (HC group, n = 11; GH group, n = 11). The volcano plot and heatmap presented in Fig. 4F,G confirmed a significant increase in IAA concentration in GH-PitNET patients.

### Relationships between serum metabolites and the clinical phenotypes of patients with GH

**Fig. 5:**
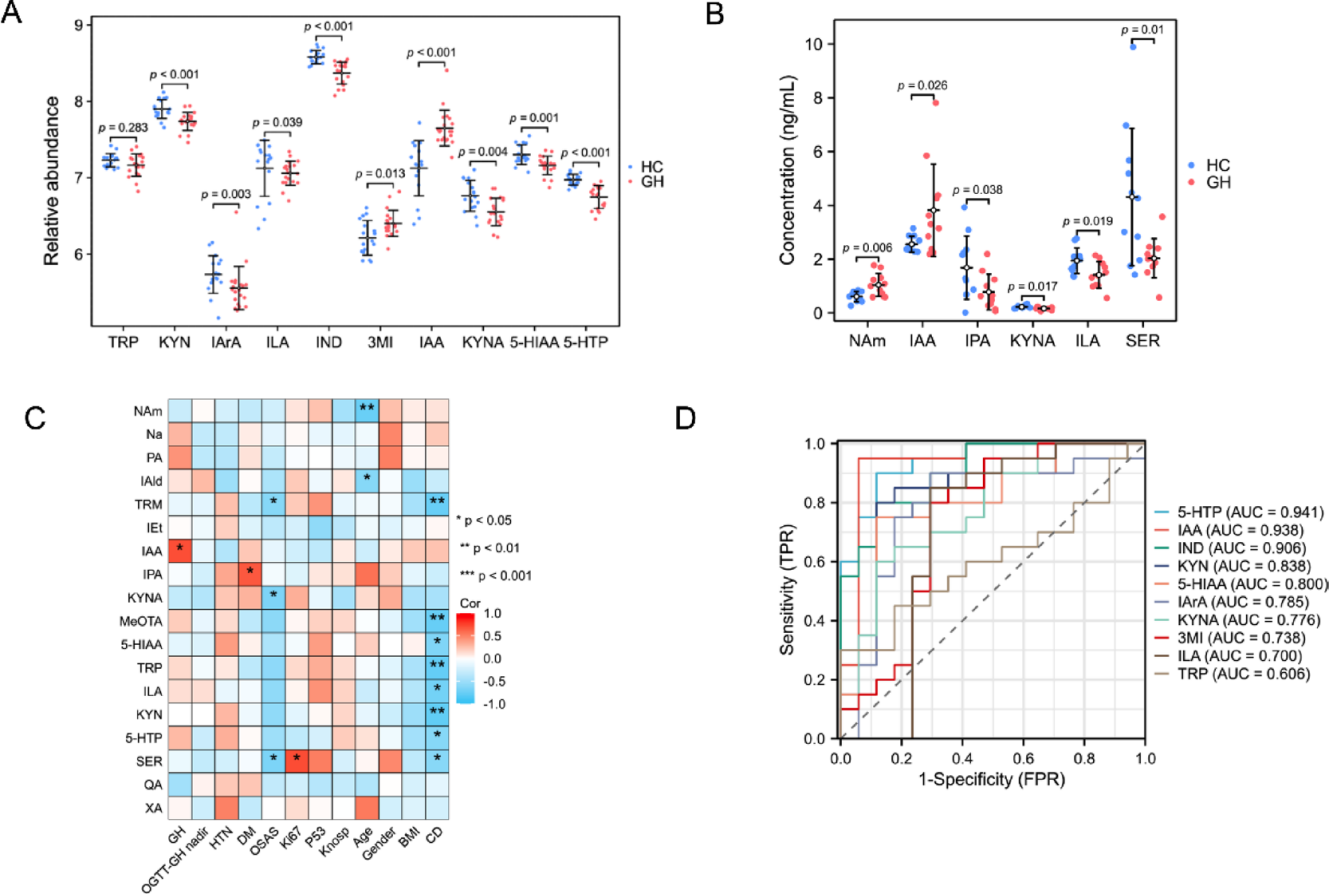
Metabolite‒clinical correlation analysis revealed that serum tryptophan metabolites were associated with the severity of clinical symptoms of GH-PitNETs. (**A**) Differences in the abundance of 10 tryptophan metabolites between the GH and HC groups. (**B**) Changes in metabolite concentrations related to tryptophan metabolism. (**C**) Correlations between tryptophan metabolites and the severity of clinical symptoms associated with GH-PitNETs. (**D**) Predictive ability of 10 tryptophan metabolites for GH-PitNETs.

We analyzed the relative abundance of differentially abundant metabolites in the tryptophan metabolic pathway using both nontargeted and targeted metabolomics. Figure 5A,B illustrates the significantly different metabolites, revealing a significant increase in IAA concentration. We further investigated the relationship between serum metabolite concentrations and clinical phenotypes, as shown in Fig. 5C. GH, a key clinical indicator, exhibited a significant positive correlation with IAA. Additionally, 5-hydroxytryptamine (5-HTP), indole-3-lactic acid (ILA), tryptophan (TRP), 5-methoxytryptamine (MeOTA), indole-3-formaldehyde (IAId), picolinic acid (PA), and nicotinic acid (Na) also showed positive correlations with GH, although these correlations did not reach statistical significance. To assess the diagnostic potential of these metabolites for GH-secreting PitNETs, we performed ROC analysis (Fig. 5D). Three metabolites, 5-HTP, IAA, and indole (IND), exhibited high diagnostic accuracy with area under the curve (AUC) values exceeding 0.9, indicating their strong predictive ability.

### Distinct patterns of bacterial, metabolite, and clinical marker cooccurring networks in GH-PitNET patients

**Fig. 6:**
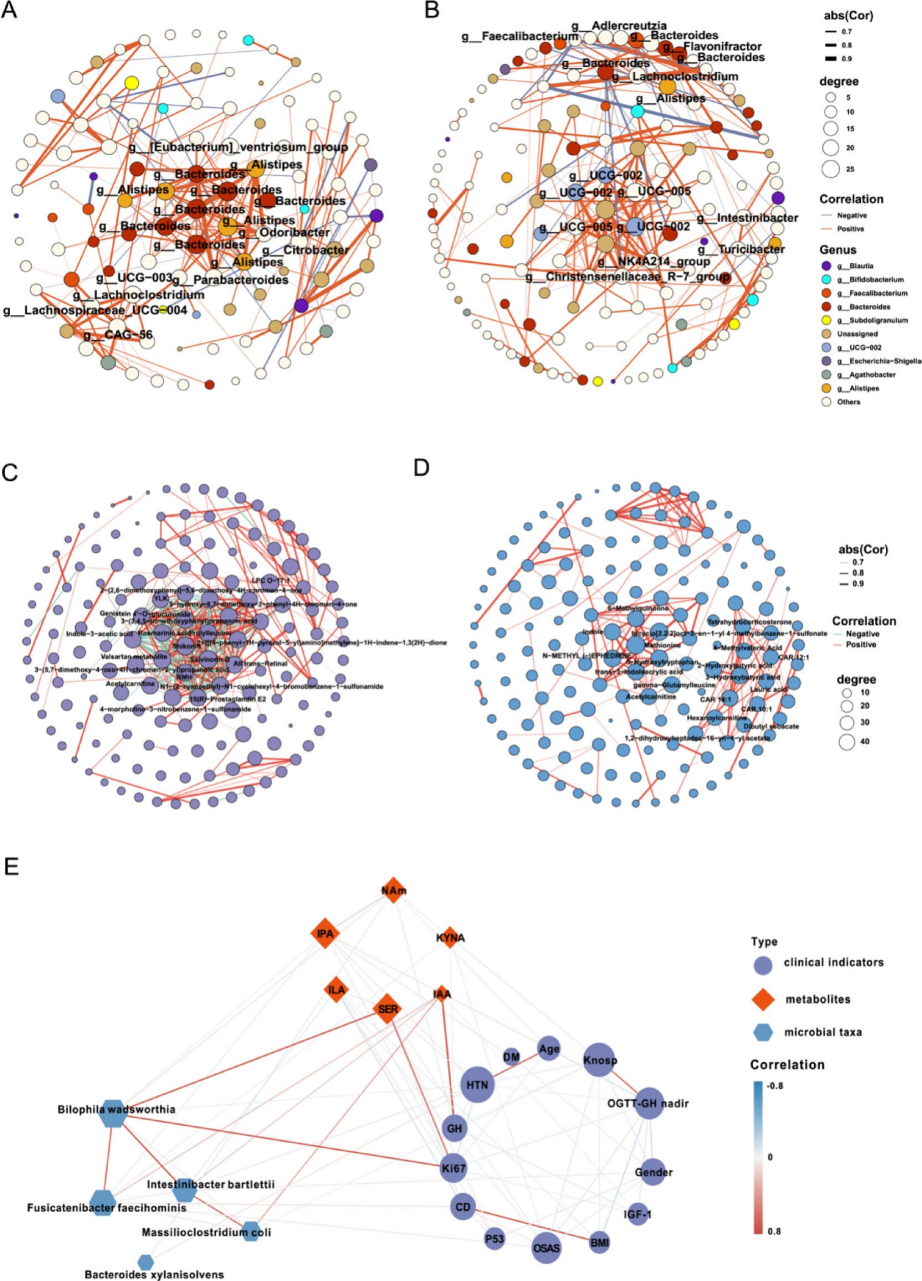
Analysis of the co-occurrence network in the HC group and GH-PitNET patients. (**A**,**B**) Cooccurrence network of gut microbial species in the HC group (**A**) and the GH group (**B**). (**C**,**D**) Serum metabolite co-occurrence network in the HC group (**C**) and GH-PitNET patients (**D**). Only the species connections (edges) > cutoff (correlation values >|0.7|, *p* < 0.05) are retained. Node size represents the degree of one genus in each network, and edge width represents the correlation value supporting the connection. Node colors represent different genus-level species, and the genus with the highest node label value is displayed. (**E**) Integrating microbiome and metabolome datasets via Cytoscape software produced a network of associations showing correlations between bacterial species, metabolites, and clinical indicators. The positive correlation between the nodes is shown by the red connecting line, and the negative correlation is shown in blue.

Owing to the dynamic nature of the gut microbiota, reflecting intricate microbial interactions within ecosystems, Spearman correlation analyses were performed on bacterial species between GH-PitNET patients and healthy controls to construct co-occurrence networks (*p* < 0.05, |correlation value|> 0.7). Figures A and B depict the co-abundance networks for the HC and GH-PitNETs groups, respectively. In the HC group, 65 bacterial-bacterial connections were identified, with species exhibiting a degree greater than 20 considered potential core species. These core species predominantly belonged to the genera *Bacteroides* and *Alistipes*. In contrast, the GH group exhibited 58 bacterial-bacterial connections, with the core species identified as *UCG-005*, *UCG-002*, and *Lachnoclostridium* (Fig. 6A,B). Similarly, Spearman correlation analyses were conducted on the serum metabolites of the HC and GH groups (*p* < 0.05, |correlation value|> 0.7). The average degree differed between the two groups, being 15.9 for HC and 11.3 for GH, suggesting a potential attenuation of metabolite interactions in GH-PitNET patients (Fig. 6C,D).

Furthermore, we investigated the interrelationships between the differential gut microbiota, metabolites, and clinical indicators. Based on the observed correlations between microbiota and clinical indicators (Fig. 3C), we identified five differential bacterial species significantly associated with GH levels. Subsequently, we constructed a correlation network involving these bacterial species, targeted metabolites, and clinical indicators (Fig. 6E). Notably, *Intestinibacter bartlettii*, *Fusicatenibacter faecihominis*, and *Massilioclostridium coli* were correlated with IAA, a metabolite also associated with GH-related clinical indicators.

### IAA contributes to GH3 cell growth hormone secretion and proliferation

**Fig. 7:**
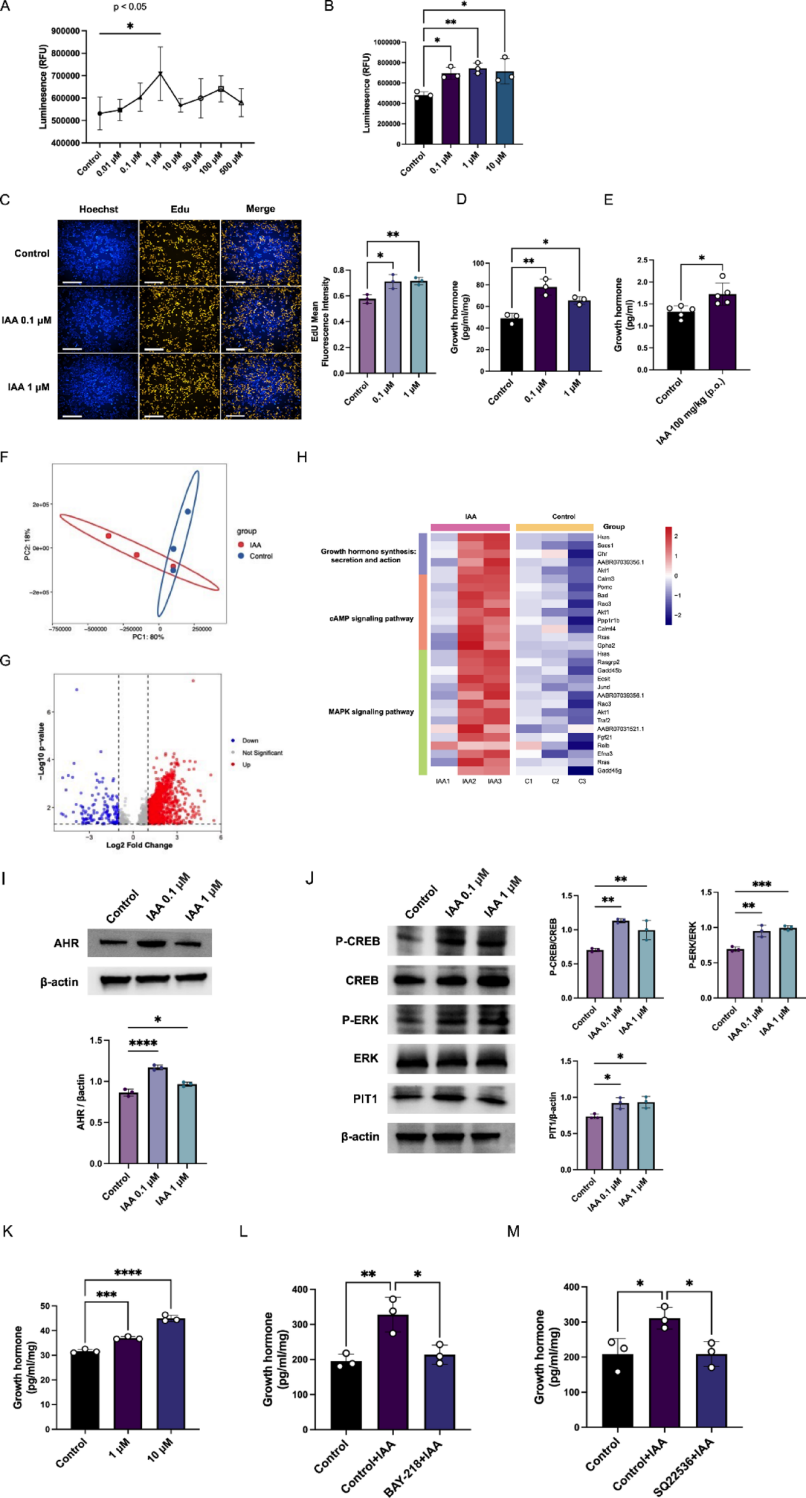
Growth hormone secretion and proliferation of GH3 cells dependent on IAA. (**A**,**B**) CellTiter-GLO (CTG) was used to detect the viability of GH3 cells after IAA treatment. A represents preliminary screening of IAA concentration, and B represents rescreening of IAA concentration, n = 3. (**C**) The percentage of EdU-positive cells was determined via fluorescence microscopy after IAA stimulation, and ImageJ was used to perform quantitative statistical analysis; n = 3; scale bar, 200 μm. (**D**) Secretion of growth hormone by GH3 cells was detected after IAA treatment, n = 3. (**E**) The changes of serum GH in mice after IAA administration for one week, n = 5. (**F**) The obtained transcriptomes were subjected to principal component analysis (PCA), n = 3. (**G**) A volcano plot was used to analyze the differential genes of the GH3 cells treated with IAA, n = 3. (**H**) Pathway enrichment of the GH3 cells after IAA treatment, n = 3. (**I**,**J**) Western blot analysis of changes in the protein expression of AHR, p-ERK1/2, ERK1/2, p-CREB, CREB, and Pit1 in GH3 cells after IAA treatment. Quantitative analysis was performed via ImageJ (n = 3). (**K**) Growth hormone secretion was detected after forskolin treatment of GH3 cells, n = 3. (**L**) Growth hormone secretion was detected after BAY-218 (10 μM) treatment for 24 h, IAA (1 μM) was treatment of GH3 cells, n = 3. (**M**) Growth hormone secretion was detected after SQ22536 (10 μM) treatment for 24 h, IAA (1 μM) was treatment of GH3 cells, n = 3.

The serum metabolomics data indicated a significant elevation in IAA levels in GH-PitNET patients, prompting the hypothesis that IAA might stimulate growth hormone secretion in these individuals. To investigate this, GH3 cells were treated with varying concentrations of IAA. As depicted in Fig. 7A, low concentrations of IAA, specifically 0.01 μM, 0.1 μM, and 1 μM, particularly 1 μM, significantly enhanced GH3 cell proliferation. Consequently, a subset of low IAA concentrations was selected for further investigation. Figure 7B confirms the proliferative effect of IAA on GH3 cells. Additionally, EdU incorporation assays were employed to further assess the impact of IAA on cell viability. As shown in Fig. 7C, IAA increased the percentage of EdU-positive cells, providing further evidence of its proliferative effect.

Next, we investigated the effect of IAA on growth hormone secretion in vivo* and vitro*. As shown in Fig. 7D,E, IAA significantly stimulated growth hormone production in GH3 cells and serum of mice. Treatment with different concentrations of IAA also significantly increased the concentration of growth hormone in the supernatant of GH3 cells (Figure S1). To further explore the underlying mechanisms, we performed RNA-sequencing analysis on IAA-stimulated GH3 cells. PCA confirmed distinct gene expression patterns in IAA-treated cells (Fig. 7F). Volcano plots were used to identify differentially expressed genes (DEGs) (Fig. 7G). Enrichment analysis revealed significant upregulation of the KEGG pathways involved in growth hormone synthesis, secretion, and action (map04935), as well as the cAMP (map04024) and MAPK (map04010) signaling pathways. These findings suggest that IAA promotes GH3 cell growth hormone secretion through activation of the cAMP signaling pathway^31^ (Fig. 7H).

We further validated these findings at the protein level. Western blot analysis demonstrated that IAA upregulated the expression of AHR, p-CREB, p-ERK1/2, and Pit1. These results indicate that IAA could activate both the cAMP and MAPK signaling pathways, thereby promoting GH3 cell proliferation and growth hormone secretion (Fig. 7I,J). To further confirm the role of the cAMP signaling pathway in growth hormone secretion, we employed the cAMP agonist forskolin, which stimulated growth hormone secretion (Fig. 7K). As shown in Fig. 6L,M. BAY-218 and SQ22536 are inhibitors of AHR and cAMP respectively. The results showed that blocking AHR and cAMP could inhibit the increase of GH secretion caused by IAA, that is, IAA up-regulated AHR protein expression. Then activation of cAMP pathway promotes GH secretion.

## Discussion

This investigation delves into the intricate relationship between the gut microbiota, serum metabolites, and clinical phenotypes in GH-PitNET patients, offering valuable insights into the underlying biological mechanisms. By analyzing a cohort of individuals in the GH and HC groups, our study elucidates the correlation between alterations in the gut microbiome and serum metabolome with the clinical manifestations of GH-PitNETs. However, these findings must be interpreted with caution due to the pilot-scale sample size, which may limit statistical power and broader applicability. Future studies with larger cohorts are warranted to validate these exploratory observations. While antibiotic/probiotic use was controlled, other factors like long-term diet (e.g., fiber intake, dietary tryptophan) and non-antibiotic medications may confound microbiota and metabolite profiles. Detailed dietary and medication histories were not systematically analyzed, limiting causal inference. Future studies should address these confounders to clarify their role in GH-PitNET pathogenesis. While our cross-sectional design identifies associations between gut dysbiosis and GH-PitNETs, causality remains unresolved. Longitudinal studies tracking microbiota/metabolite dynamics from pre-tumor stages are critical to establish whether microbial shifts drive tumor progression or are secondary to hormonal/metabolic changes.

The observed differences in gut microbiota composition between the GH and HC groups are crucial for understanding the broader metabolic implications of GH-PitNETs. The higher prevalence of diabetes mellitus and significantly greater BMI in the GH group may contribute to these alterations in gut microbiota composition. While the dominance of *Firmicutes* and *Bacteroides* is consistent with the general composition of the human gut microbiota, their relative proportions were significantly altered in the GH group^32–37^. This shift may reflect metabolic imbalances associated with GH pathology, as these phyla are linked to various metabolic functions and disorders^38–40^. The predominance of specific genera, such as *Bacteroides*, in the GH group may be indicative of an altered metabolic environment. These bacteria play pivotal roles in carbohydrate and bile acid metabolism, processes that are likely disrupted in the context of elevated GH levels^41–44^. Therefore, the gut microbiome composition of GH-PitNET patients not only reflects the metabolic disturbances associated with the disease but may also contribute to its progression and exacerbation.

Our study, focused on serum metabolomics, identified distinct metabolic profiles between individuals with GH-PitNETs and HC, with particular emphasis on tryptophan metabolism in the GH-PitNETs group. Given the extensive role of tryptophan metabolites in immune function and neurotransmitter balance^45–48^, these alterations may reflect the systemic impact of GH hypersecretion on various physiological pathways. The potential of specific metabolites, including 5-hydroxytryptophan, indole-3-acetic acid, and indole, to serve as biomarkers for GH-PitNETs is a significant finding. The robust diagnostic accuracy indicated by the ROC analysis underscores their potential utility in early detection of this condition, a critical factor in improving patient outcomes.

The gut microbiota is intricately linked to host metabolism, endocrinology, and pathophysiology^49^. By analyzing diverse microbial taxa and their associated metabolites, key associations were identified between GH-positive and GH-negative taxa, as well as disease severity. Both bacterial and metabolic networks differed between the HC and GH groups, exhibiting variations in network density, core species, and pivotal metabolites. The study highlighted bacteria that metabolize tryptophan into indole derivatives, including *anaerobic rods*, *Bacteroides*, *Clostridia*, *Bifidobacterium*, and *lactobacilli*^50^. For instance, several *Bacteroides* and *Clostridium bartlettii* species produce IAA and ILA, while *Bifidobacterium spp*. produce ILA^51^. In our research, Intestinibacter *bartlettii*, *Fusicatenibacter faecihominis*, and *M. coli* were positively correlated with IAA and promoted GH secretion. *Bilophila wadsworthia*-mediated relationships were observed between microbial taxa and the cell proliferation marker Ki67. These findings underscore the potential therapeutic strategies targeting the gut microbiota and its metabolites for patients with GH-PitNETs.

Cancer cells are characterized by metabolic dysfunction and remodeling^52–55^. Amino acids, such as arginine and tryptophan, are metabolized through various pathways to support cell proliferation and anabolic growth^56^. Among the predicted metabolic pathways, we primarily explored tryptophan metabolism. The tryptophan metabolic pathway primarily encompasses three pathways: the kynurenine (Kyn) pathway, the 5-hydroxytryptamine (5-HTP) pathway, and the indole pathway^57^. The kynurenine pathway is primarily associated with inflammation, immune response, and excitatory neurotransmission, while the 5-HTP pathway is linked to neurotransmitter secretion^58,59^. The indole pathway, closely associated with gut microbiota, directly converts tryptophan into various molecules, including IAA, IPA, and indole-3-aldehyde (3-IAld)^60^. Indole and its derivatives maintain intestinal homeostasis by regulating pro- and anti-inflammatory cytokines^50^. Additionally, they contribute to gastrointestinal function, inflammation, and antioxidant and immune system regulation^61^.

A recent study noted that elevated tryptophan metabolites like indole 3-pyruvate derivatives can exacerbate tumor development^62^. This resonates with the observed elevation of IAA in GH-PitNETs and its potential role in promoting GH secretion through the cAMP pathway, suggesting that indole derivatives might represent a broader tumor-promoting pathway. Studies in pancreatic cancer suggest that tryptophan-derived metabolites can activate the aryl hydrocarbon receptor (AHR) in tumor-associated macrophages, leading to reduced anti-tumor immunity^63^. In GH-PitNETs, the elevated IAA may act in a similar way, potentially activating AHR and creating an environment that supports tumor growth by weakening immune defenses. This link suggests that microbial metabolites like IAA may play a dual role in GH-PitNETs—directly increasing tumor cell growth and indirectly promoting tumor progression by dampening immune responses.

Previous studies have indicated that tryptophan, an endogenous metabolically selective amine and IAA, directly binds to the aryl hydrocarbon receptor (AHR)^64^. AHR, a ligand-activated transcription factor, influences immune cell function and development. Notably, the AHR pathway has been implicated in the biological processes underlying GH-PitNETs^65^. The aromatic receptor-cAMP-phosphodiesterase pathway is a significant contributor to mutations in the aromatic receptor-interacting protein (AIP), leading to pituitary tumor formation. Furthermore, somatic activating mutations in the α subunit of stimulatory G proteins (GNAS, or gsp oncogenes) are detected in 30–40% of GH-secreting pituitary tumors^14^. Gain-of-function mutations in GαS can trigger the development of pituitary tumors and other cAMP-dependent tumors, leading to growth hormone secretion. In this study, we explored the relationship between tryptophan metabolism and growth hormone secretion. Our findings confirmed that IAA can dysregulate the expression of AHR and activate the cAMP pathway, stimulating growth hormone secretion in GH3 cells.

Given that all participants in this study were Han Chinese, there is a potential limitation in the generalizability of our findings to other ethnic populations, who may have distinct gut microbiota profiles due to genetic, environmental, and dietary differences. Future studies are recommended to investigate the microbiota composition in more ethnically diverse cohorts to validate and extend these findings. While this study shows an association between gut microbiota changes and GH-PitNETs progression, only a longitudinal study could clarify whether these changes cause or result from tumor growth. Following patients over time would give clearer insights into how gut microbiota might influence GH-PitNETs. The relationship between microbial derived IAA and GH secretion also needs further exploration. Another important consideration in our findings is the potential influence of confounders, such as dietary differences and individual microbiome variability. While we minimized these effects by selecting participants with similar diets, diet remains a significant factor affecting microbiome composition. Additionally, individual microbiome variability could impact the results. Future studies should more rigorously control dietary intake and investigate how microbiome differences influence disease progression in growth hormone-secreting PitNETs to strengthen the robustness of these findings.

In conclusion, the findings of this study offer a novel insight into the underlying mechanisms of tumor progression and hormone secretion in GH-PitNETs. A deeper understanding of the interplay between gut microbiota and serum metabolites in this context could inform more targeted therapeutic strategies, potentially leading to improved patient outcomes. The potential utility of these biological entities as biomarkers for early detection and prognostication offers promising avenues for clinical practice. Our data suggest gut microbiota dysbiosis in GH-PitNET patients may contribute to elevated IAA levels and GH hypersecretion via cAMP signaling. While microbial taxa like *Intestinibacter bartlettii* correlate with IAA, causation remains unproven. Future studies should validate these associations using in vivo models or microbial transplantation to confirm mechanistic links.

## Electronic supplementary material

Below is the link to the electronic supplementary material.

Supplementary Material 1

Supplementary Material 2

## Data Availability

All raw 16S rRNA sequencing data generated in this study have been deposited in the NCBI Sequence Read Archive (SRA) under BioProject accession number PRJNA1244248. The metabolomics datasets are publicly available in the MetaboLights repository under accession number MTBLS12341.

## Abbreviations

GH-PitNETs: Growth hormone-secreting pituitary neuroendocrine tumors
GH: Growth hormone
IAA: 3-Indoleacetic acid
IGF-1: Insulin-like growth factor-1
OGTT-GH nadir: Growth hormone nadir during the oral glucose tolerance test
PCA: Principal component analysis
PCoA: Principal coordinate analysis
CTG: CellTiter-Glo
5-HTTP: 5-Hydroxytryptamine
ILA: Indole-3-lactic acid
MeOTA: 5-Methoxytryptamine
IAId: Indole-3-formaldehyde
PA: Picolinic acid
NA: Nicotinic acid
