# Supplementary material for: Multi-omics study reveals gut microbiota dysbiosis and tryptophan metabolism alterations in GH-PitNET progression
Source: Sci Rep. 2025 Jul 7;15:24261. doi: 10.1038/s41598-025-07812-x (PMC12234824; doi:10.1038/s41598-025-07812-x)
Supplement: Supplementary file 1 — Supplementary Material 1 [file 41598_2025_7812_MOESM1_ESM.pdf]

Repeat 1

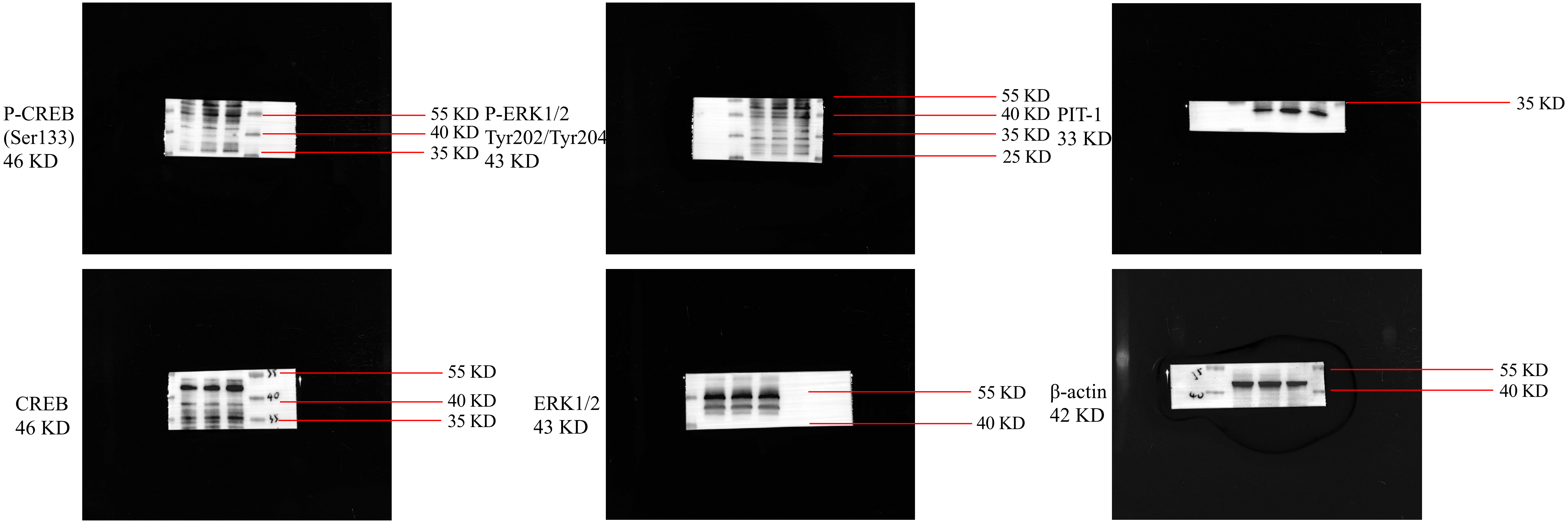

Lane 1: Control (DMSO)

Lane 2: IAA 0.1 μM

Lane 3: IAA 1 μM

Repeat 2

P-CREB (Ser133) 46 KD

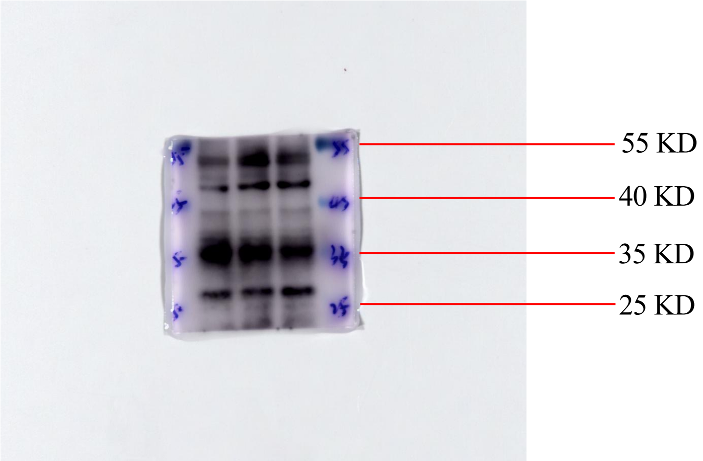

P-ERK1/2 Tyr202/Tyr204 43 KD

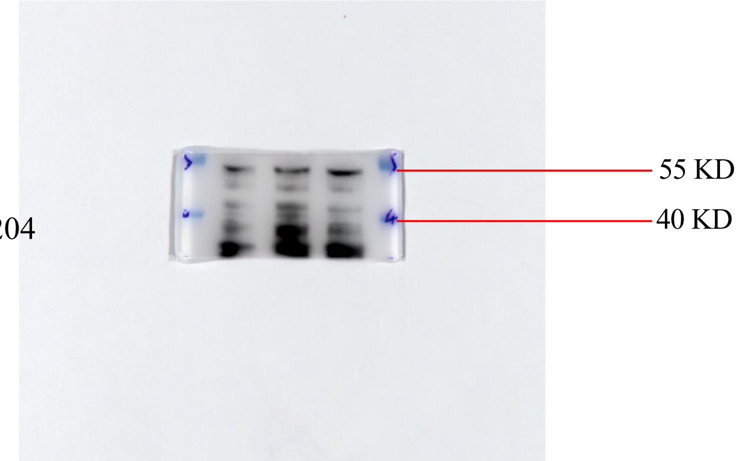

PIT-1 33 KD

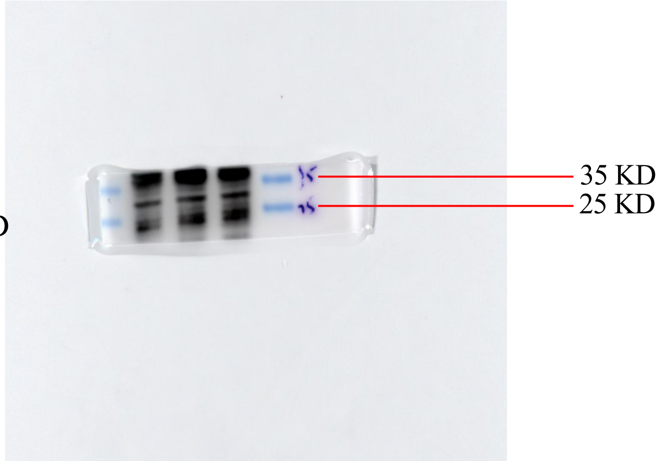

CREB 46 KD

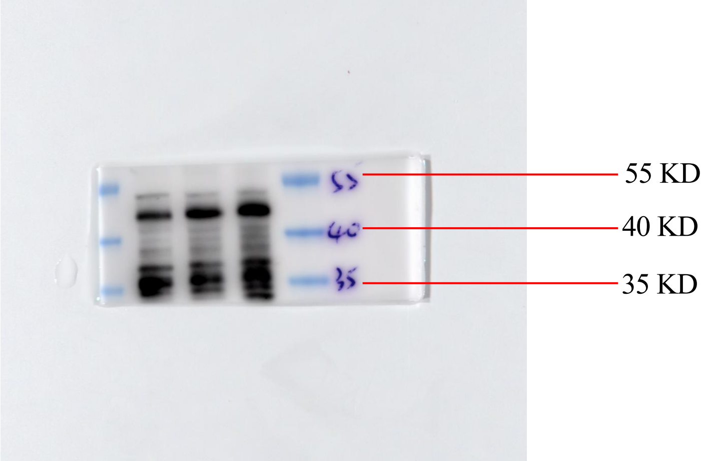

ERK1/2 43 KD

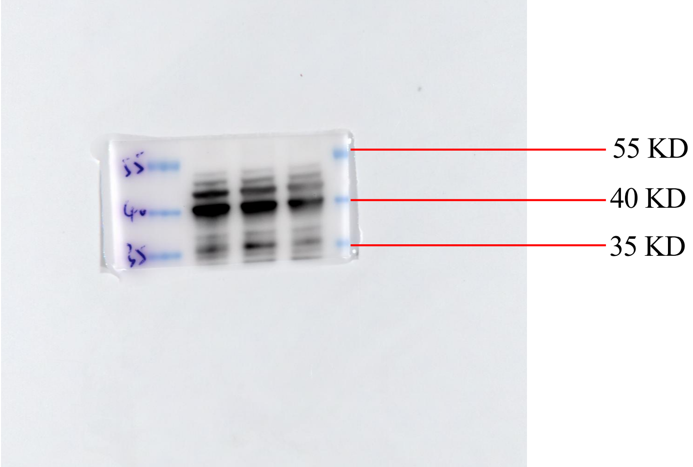

β-actin 42 KD

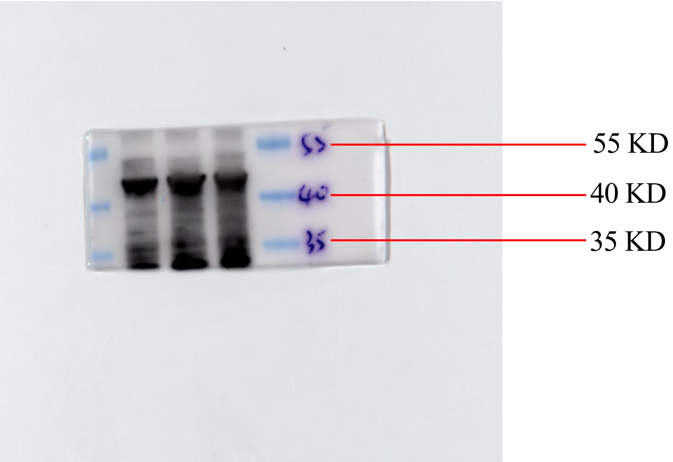

Lane 1: Control (DMSO)

Lane 2: IAA 0.1 μM

Lane 3: IAA 1 μM

Repeat 3

P-CREB
(Ser133)
46 KD

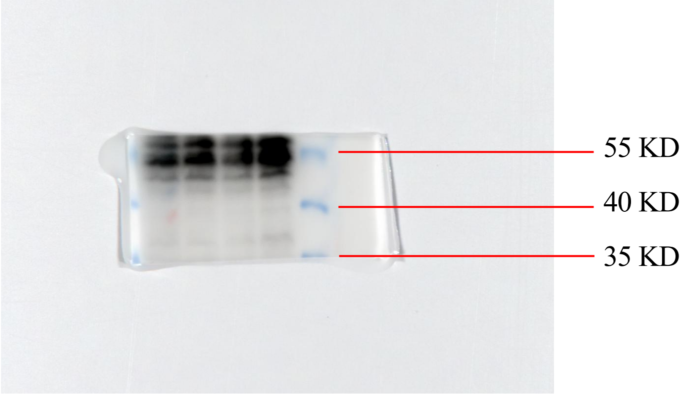

P-ERK1/2
Tyr202/Tyr204
43 KD

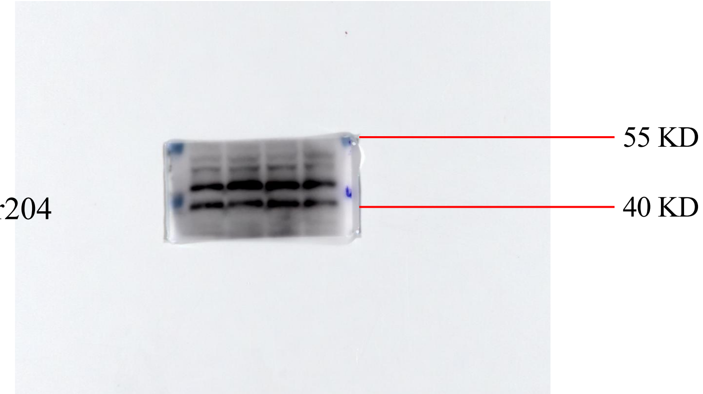

PIT-1
33 KD

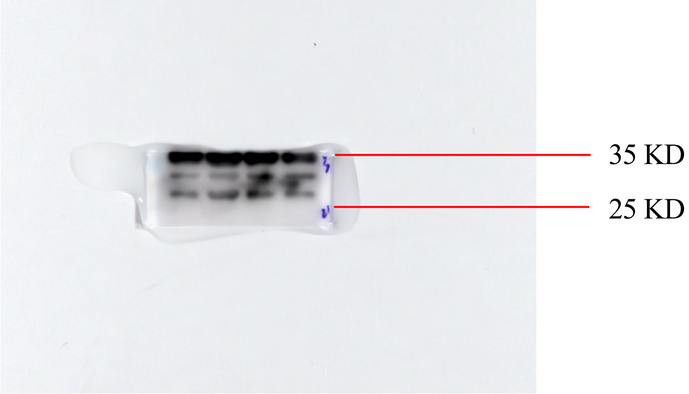

CREB
46 KD

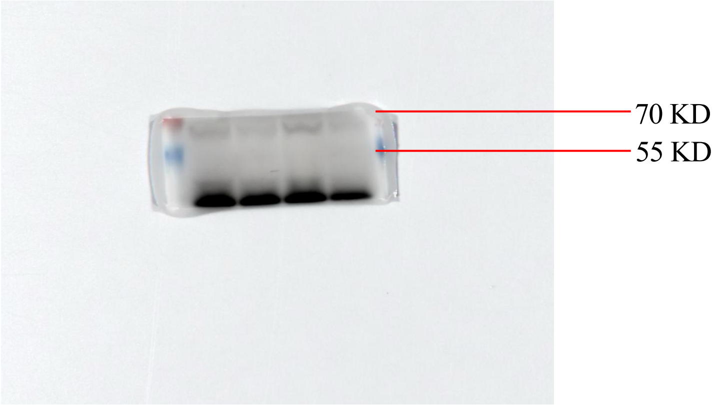

ERK1/2
43 KD

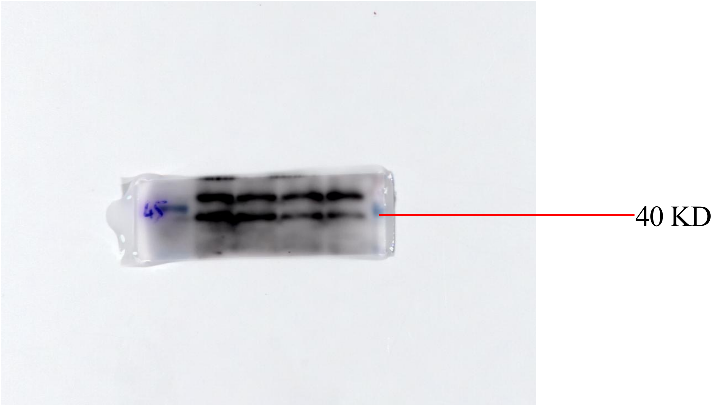

β-actin
42 KD

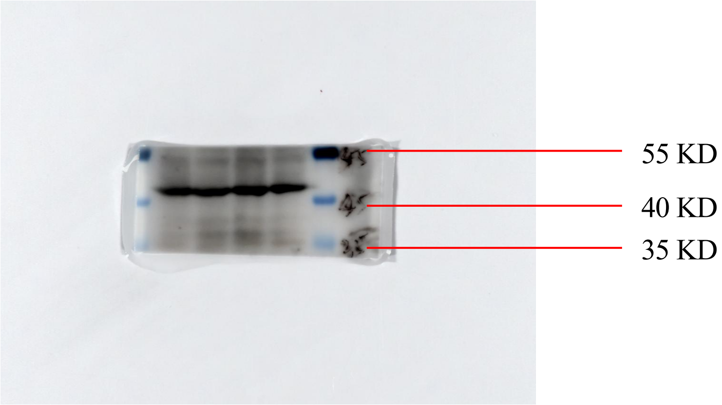

Lane 1: Control (DMSO)

Lane 2: IAA 0.1 μM

Lane 3: IAA 1 μM

Lane 4: IAA 10 μM

AHR repeat

Repeat 1

AHR
110 KD

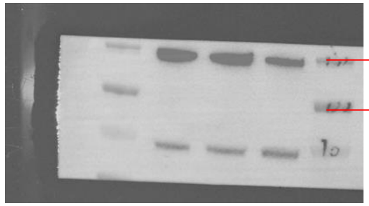

130 KD
100 KD

B-actin
42 KD

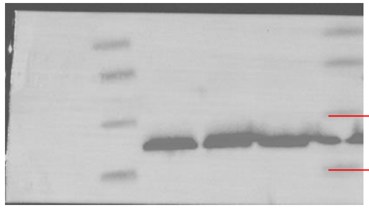

55 KD
40 KD

Repeat 2

AHR
110 KD

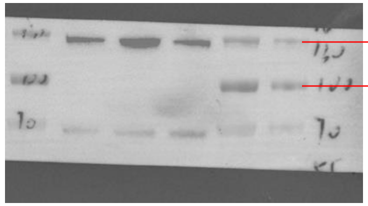

130 KD
100 KD

B-actin
42 KD

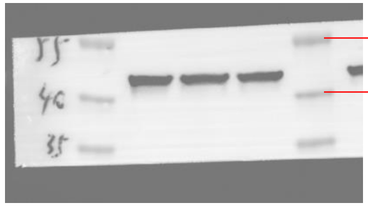

55 KD
40 KD

Repeat 3

AHR
110 KD

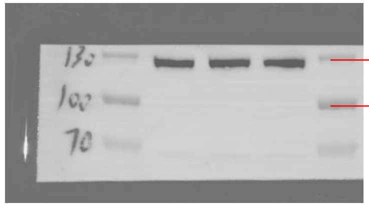

130 KD
100 KD

B-actin
42 KD

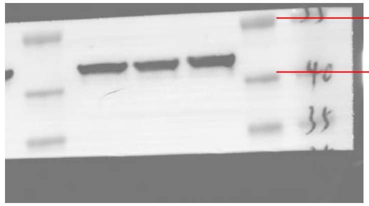

55 KD
40 KD
