# Supplementary material for: Multi-omics study reveals gut microbiota dysbiosis and tryptophan metabolism alterations in GH-PitNET progression
Source: Sci Rep. 2025 Jul 7;15:24261. doi: 10.1038/s41598-025-07812-x (PMC12234824; doi:10.1038/s41598-025-07812-x)
Supplement: Supplementary file 2 — Supplementary Material 2 [file 41598_2025_7812_MOESM2_ESM.docx]

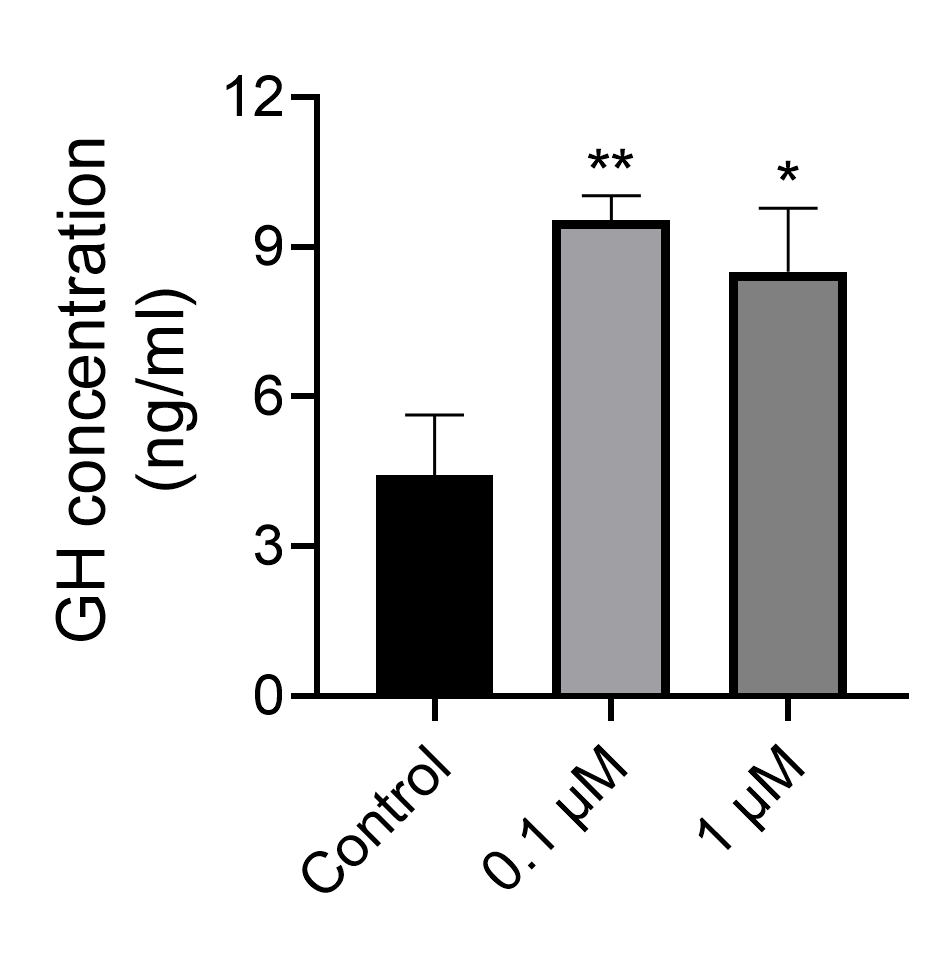


**Supplementary Figure 1. Growth hormone concentration in supernatant of GH3 cells was detected after IAA treatment, n=3.**
